# Integrative analysis of genome and transcriptome reveal the genetic basis of high temperature tolerance in pleurotus giganteus (Berk. Karun & Hyde)

**DOI:** 10.1186/s12864-023-09669-8

**Published:** 2023-09-18

**Authors:** Yang Yang, Yongru Pian, Jingyi Li, Lin Xu, Zhu Lu, Yueting Dai, Qinfen Li

**Affiliations:** 1 Environment and Plant Protection Institute, Chinese Academy of Tropical Agricultural Sciences, Haikou, China; 2 Engineering Research Center of Chinese Ministry of Education for Edible and Medicinal Fungi, Jilin Agricultural University, Changchun, China; 3 Key Laboratory of Low Carbon Green Agriculture in Tropical China, Ministry of Agriculture and Rural Affairs, Haikou, P. R. China; 4 National Agricultural Experimental Station for Agricultural Environment, Danzhou, China; 5 Jilin Academy of Vegetables and Flowers Sciences, Changchun, China

**Keywords:** Zhudugu, High temperature stress, Genome, Transcriptome analysis, Heat shock protein, Heat signal transduction, qPCR

## Abstract

**Background:**

*Pleurotus giganteus* is a commonly cultivated mushroom with notable high temperature resistance, making it significant for the growth of the edible fungi industry in the tropics. Despite its practical importance,, the genetic mechanisms underlying its ability to withstand high temperature tolerance remain elusive.

**Results:**

In this study, we performed high-quality genome sequencing of a monokaryon isolated from a thermotolerant strain of *P*. *giganteus*. The genome size was found to be 40.11 Mb, comprising 17 contigs and 13,054 protein-coding genes. Notably, some genes related to abiotic stress were identified in genome, such as genes regulating heat shock protein, protein kinase activity and signal transduction. These findings provide valuable insights into the genetic basis of *P. giganteus’* high temperature resistance. Furthermore, the phylogenetic tree showed that *P. giganteus* was more closely related to *P. citrinopileatus* than other *Pleurotus* species. The divergence time between *Pleurotus* and *Lentinus* was estimated as 153.9 Mya, and they have a divergence time with *Panus* at 168.3 Mya, which proved the taxonomic status of *P. giganteus* at the genome level. Additionally, a comparative transcriptome analysis was conducted between mycelia treated with 40 °C heat shock for 18 h (HS) and an untreated control group (CK). Among the 2,614 differentially expressed genes (DEGs), 1,303 genes were up-regulated and 1,311 were down-regulated in the HS group. The enrichment analysis showed that several genes related to abiotic stress, including heat shock protein, DnaJ protein homologue, ubiquitin protease, transcription factors, DNA mismatch repair proteins, and zinc finger proteins, were significantly up-regulated in the HS group. These genes may play important roles in the high temperature adaptation of *P. giganteus*. Six DEGs were selected according to fourfold expression changes and were validated by qRT-PCR, laying a good foundation for further gene function analysis.

**Conclusion:**

Our study successfully reported a high-quality genome of *P. giganteus* and identified genes associated with high-temperature tolerance through an integrative analysis of the genome and transcriptome. This study lays a crucial foundation for understanding the high-temperature tolerance mechanism of *P. giganteus*, providing valuable insights for genetic modification of *P. giganteus* strains and the development of high-temperature strains for the edible fungus industry, particularly in tropical regions.

**Supplementary Information:**

The online version contains supplementary material available at 10.1186/s12864-023-09669-8.

## Introduction

*Pleurotus giganteus* (Berk.) Karunarathna & K.D. Hyde, commonly known as zhudugu, is an esteemed edible mushroom renowned for its delectable taste and promising commercial potential [1, 2]. Furthermore, *P. giganteus* has the advantages of robust environmental adaptability, uncomplicated cultivation technology and high biological conversion rate, which has gradually earned the attention of edible fungi producers, and the cultivation scale has been continuously expanded [3–6]. In particular, *P. giganteus* can tolerate high temperature during the whole growth and development period, which makes it play a significant role in regulating the market supply of mushrooms and off-season cultivation. The annual average temperature in tropical areas is relatively high, but most edible fungi belong to medium-low temperature type, and there are few edible fungi suitable for large-scale cultivation in tropical areas. Therefore, the emergence of *P. giganteus* also provides new opportunities for the industrial development of edible fungi in the tropics [7].

In recent years, the impacts of global warming and frequent high temperature conditions have become increasingly serious. As a crucial environmental factor, temperature directly influences the metabolism and physiological activities of organisms [8, 9]. In edible fungi, unsuitable temperatures can slow down hyphae growth of and inhibit the formation of fruiting bodies, ultimately affecting their quality, yield, and economic value [10, 11]. Extreme high temperature (HT) can lead to increased nutrient metabolism and may even cause death [12]. Our previous research has shown that after being subjected to high temperature stress, *P. giganteus* will have problems such as reduced marketability, prolonged growth period, increased disease susceptibility, and even failure to produce mushroom, which has brought significant challenges to producers. Therefore, it is thus crucial to screen high-temperature resistant strains of *P. giganteus* and reveal their molecular regulatory mechanisms for high-temperature environmental adaptability.

Multi-omics analysis serves as a fundamental approach for identifying key genes and analyzing their functions, playing a crucial role in exploring the growth and development of organisms and their adaptation to adversity [13, 14]. The application of genomics combined with transcriptome technologies to analyze the complex molecular mechanisms of biological adaptation to the environment is a current research focus [15, 16]. Previous studies have demonstrated that after exposure to temperature stress, a large number of genes in plants and animals are differentially regulated, and these genes are involved in biological processes including cell growth and differentiation, transcriptional regulation, and immune response [17–19]. However, while the cultivation techniques of *P. giganteus* are well understood, our knowledge of the relevant genetic regulation under rather high temperatures conditions remains limited. Understanding how *P. giganteus* regulates its biological processes in response to high temperatures at the genetic level is still elusive.

In this study, we focus on the high-temperature tolerance of *P*. *giganteus* mycelia, as it plays a crucial role in determining whether the strain can thrive in a high temperature environment, ultimately influencing fruiting body production. To achieve this, we utilized the Pacific Biosciences (PacBio) HiFi sequencing method to assemble a new, high-quality genome of a high-temperature tolerant strain of *P. giganteus*. Additionally, we performed a comprehensive transcriptome analysis to explore expression differences between mycelia subjected heat treatment and control groups. Our primary objectives were twofold: to present a high-quality genome of *P. giganteus* and conduct an in-depth analysis of its genome components and gene functions. Secondly, we aimed to screen key tolerance genes and explore the genetic basis of stress response to high temperature. By achieving these objectives, our work provides valuable insight into the molecular mechanism underlying high-temperature response and tolerance in *P. giganteus*, while also paving the way for targeted genetic improvement of *P. giganteus*.

## Results

### Genome sequencing and assembly of *P. giganteus*

Based on the PacBio HiFi sequencing method, the whole genome sequencing of *P. giganteus* PG46 produced 8.78 Gb of sequence data, representing 478,078 subreads. *De novo* assembly results showed that the total sequence length of the *P. giganteus* PG46 genome was 40.11 Mb, comprising 17 contigs with a 2.89 Mb N50 value, and the guanine-cytosine content (GC content) was 50.45% (Table 1). Furthermore, high-quality standards were met, with CEGMA and BUSCO values reaching 97.65% and 95.90% respectively, confirming the reliability of the PG46 genome assembly.

**Table 1:** Genome assembly and annotation statistics of *P. giganteus*

Accession	*P. giganteus* (PG46)
Genome size (Mb)	40.11
Number of contigs	17
N50 (Mb)	2.89
GC Content (%)	50.45
Gene Number	13,054
Gene average length (bp)	1,462
CEGMA (%)	97.65
BUSCO (%)	95.90

### Gene prediction and functional annotation

**Fig. 1:**
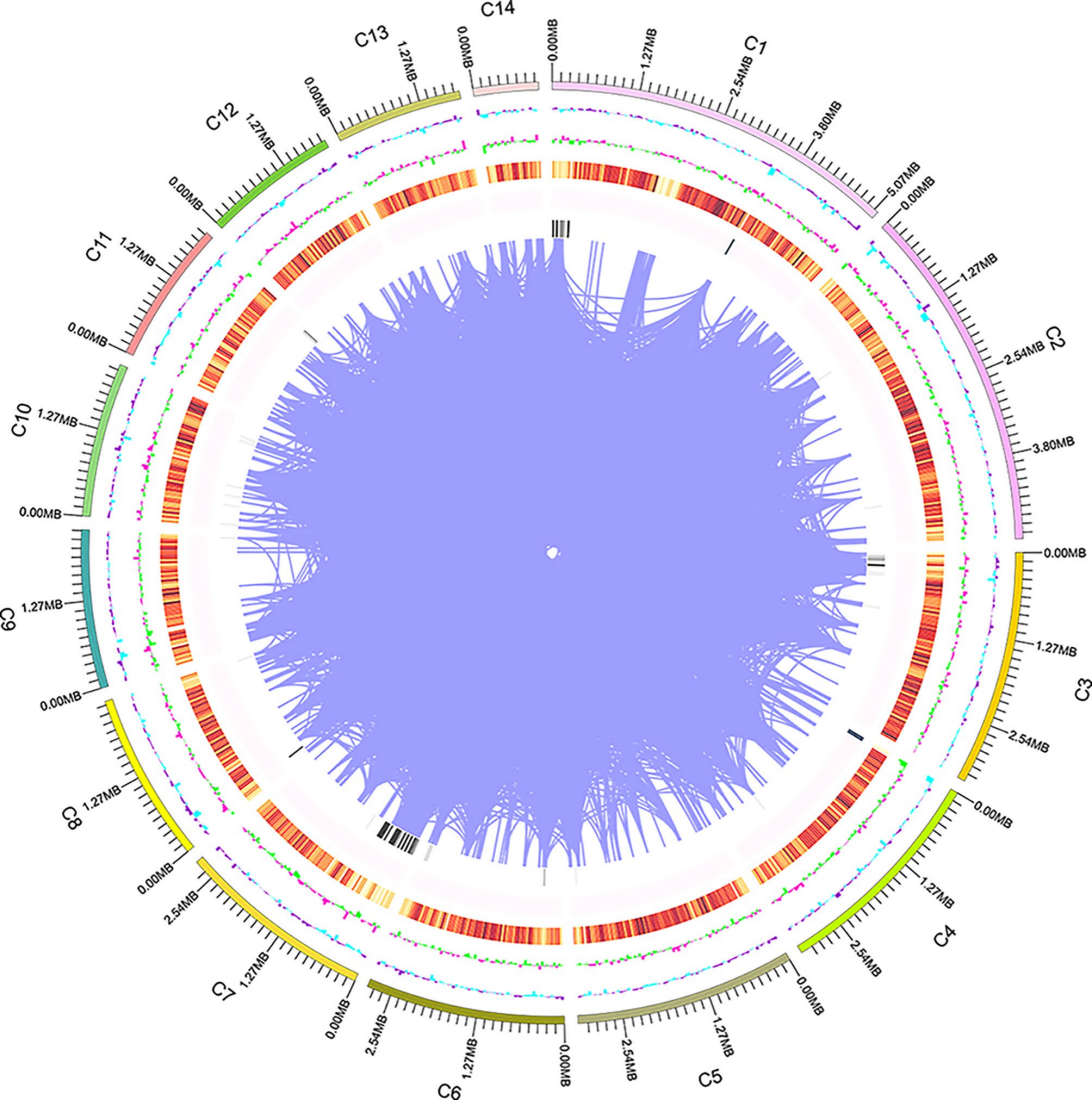
The genome map of *P. giganteus*. Outside to inside of concentric circles show GC content, assembly scaffold number, gene density, non-coding RNA (ncRNA), all repeat content, LTR content, LINE content, DNA repeat content, satellite content

A total of 13,054 protein-coding genes with an average sequence length of 1,462 bp were predicted in the *P. giganteus* PG46 genome by the combination of transcriptome prediction and homologous genewise prediction. To visually represent the genome characteristics, a genetic map of *P. giganteus* PG46 was drawn (Fig. 1). For non-coding RNA, only 830 tRNAs and 21 rRNAs were annotated based on *de novo* prediction. Furthermore, scattered repeats and tandem repeats were detected in the PG46 genome, accounting for 2.02% and 0.75% of the genome size, respectively. Among the scattered repeats, LTRs were the predominant type, followed by DNA, LINE, SINE, and RC, with 17 sequences remaining unidentified. In the tandem repeat sequence, there were 3,382 minisatellite DNAs and 569 microsatellite DNAs.

**Fig. 2:**
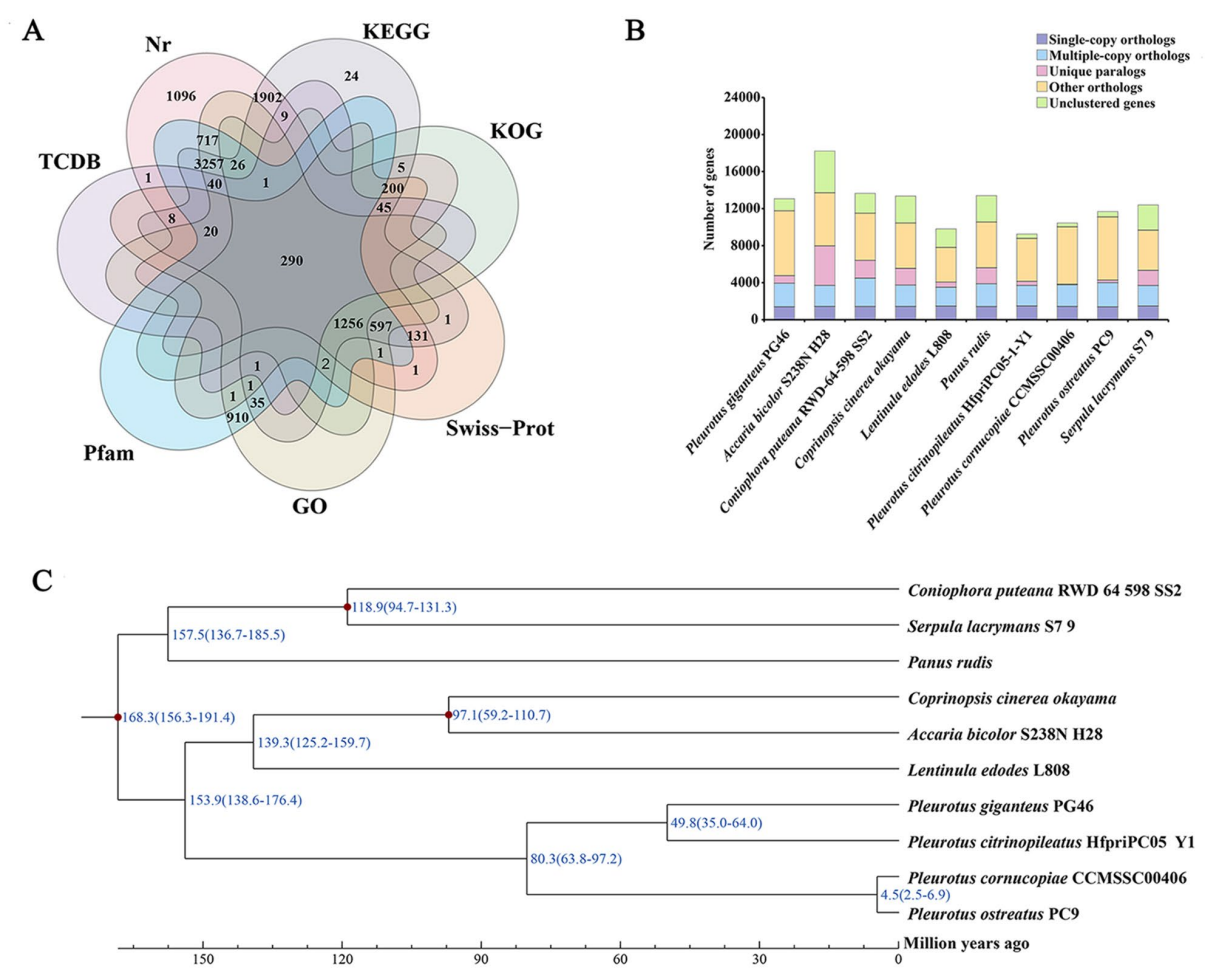
The function annotation and Phylogenetic analysis of PG46. **(A)** The function annotation of *P. giganteus*; **(B)** Comparison of orthologous genes among the genomes of 10 fungal species; **(C)** Phylogenetic tree and divergence time of *P. giganteus* PG46

Among the 13,054 coding genes, 9,603 genes (73.56%) were matched to the Nr database, followed by the KEGG database (7,851, 60.14%), GO database, Pfam database (7,155, 54.81%), SwissProt database (2,555, 19.57%), KOG database (1,825, 13.98%) and TCDB database (415, 3.18%) (Fig. 2A). For GO annotation, 7,155 annotated genes were categorized into three major ontologies and 47 functional classes (Supplementary Figure S1A). Notably, some functional genes related to abiotic stress were annotated, including DNA repair, heat shock protein binding, transmembrane transport, zinc ion binding, protein kinase activity, and signal transduction. These genes may play important roles in the adaptation of *P. giganteus* to high temperature. Regarding KEGG annotation, 371 pathways were identified, primarily distributed across cellular processes, environmental information processes, genetic information processes, metabolic processes, and organismal systems (Supplementary Figure S1B). The pathways with prominent gene annotations included global and overview maps, translation, carbohydrate metabolism, signal transduction, transport, and catabolism. In addition, several pathways related to abiotic stress were predicted, including mTOR, MAPK, PI3K-Akt, AMPK, Hedgehog and Rap1 signal pathways, offering valuable insights into the high temperature resistance mechanisms of *P. giganteus*.

In addition, a total of 646 CAZymes genes were annotated in PG46 genome, including 248 GHs, 125 AAs, 96 CBM, 95 GTs, 56 CE and 26 PLs. Among them, numerous genes associated with lignocellulose synthesis, modification, and degradation were annotated, involving 110 AAs (AA3, AA9, AA5, AA1, AA2), 59 GHs (GH3, GH18, GH16, GH7, GH53), 64 CBMs (CBM1, CBM13) and 10 CEs (CE15, CE4, CE8). These findings suggest that PG46 may has a powerful lignocellulose degradation capacity. Furthermore, the analyses of secondary metabolites, secretory proteins and CYPs also were useful measure for functional genes prediction. In the *P*. *giganteus* PG46 genome, some important secondary metabolism gene clusters, like two type 1 polyketide synthase clusters (T1PKS), one non-ribosomal peptide synthases cluster (NRPSs), 11 non-ribosomal peptide synthases-like clusters (NRPSs-like), and one hybrid cluster of NRPSs-like and T1PKS (NRPSs-like, T1PKS) were predicted, which can mediate the synthesis of raw materials (polyketide and non-ribosomal peptide compounds) for some important drugs, providing potential opportunities for medicinal research of *P. giganteus*. Moreover, the localization detection of predicted secreted proteins showed that 1,152 proteins contained signal peptide structures, 2,130 proteins contained transmembrane structures, and 858 proteins contained both structures. Additionally, a total of 205 CYPs were identified in PG46 genome, including 167 E-class P450, 10 Cytochrome P450 and 20 undetermined P450s that may be involved in the synthesis of bioactive components, nutrients acquisition, and adaptation to environmental stress.

### Phylogenetic and evolutionary analysis of *P. giganteus*

To understand the genetic relationship of *P*. *giganteus* with other fungal species, we conducted a phylogenetic analysis using the proteomes of PG46 and nine other fungal species. The OrthoMCL clustering method identified 12,612 gene families (Fig. 2B) with 29 (*P. cornucopiae*) – 1,189 (*La. bicolor*) unique gene families among the species. Additionally, 1,053 single-copy orthologous genes were annotated among the ten species. These single-copy orthologous genes were used for phylogenetic tree construction by the maximum likelihood method to analyze the genetic relationship of *P. giganteus* with other *Pleurotus* species. The phylogenetic analysis showed that four *Pleurotus* species clustered together on one branch. *P. gigantea* was closer to *P. citrinopileatus*, and they formed a small clade. The divergence time between *P. ostreatus* and *P. cornucopiae* was estimated as ~ 4.5 (2.5–6.9) Mya, while the divergence time between *P. giganteus* and *P. citrinopileatus* was estimated as ~ 49.8 (35.0–64.0) Mya (Fig. 2C). In addition, the divergence time between *Pleurotus* and *Le. edodes* was estimated as ~ 153.9 (138.6–176.4) Mya, and they have a divergence time with *Pa. rudis* at ~ 168.3 (156.3–191.4) Mya. The results indicated that zhudugu belongs to *Pleurotus*, and not to *Panus* or *Lentinus*.

### RNA-sequencing and assessment of the sequencing results

In order to understand how *P*. *giganteus* responds to heat stress at the transcriptional level, six cDNA libraries were constructed using samples subjected to 40 °C heat stress for 18 h (HS1, HS2, HS3) and 0 h (CK1, CK2, CK3). A total of 30.9–33.16 million raw reads were generated based on the Illumina Hiseq platform. After quality evaluation and trimming, 29.09–32.60 million clean reads were obtained, and the average values of Q20, Q30, and GC contents were 96.48%, 90.84%, and 52.00%, respectively. Moreover, 72.07–81.03% of the clean reads could be mapped to the reference genome of *P. giganteus* (Table 2). Then, all the obtained RNA sequences were assembled by StringTie software, and 13,767 transcripts and 4,767 genes were obtained. Among these, 544, 464, and 620 genes were annotated by aligning the sequences to the GO, KEGG and Pfam databases, respectively.

**Table 2:** Sequence data and mapping results of *P. giganteus* genome

Samples	Raw reads (bp)	Clean reads (bp)	Q20 (%)	Q30 (%)	GC content (%)	Mapping rate (%)
CK1	30,992,442	30,955,730	97.53	93.17	51.59	81.03
CK2	31,419,736	30,707,824	96.08	90.01	52.36	78.27
CK3	30,916,202	29,893,582	96.11	89.91	52.27	78.00
HS1	33,162,848	32,595,078	96.91	91.74	51.93	73.69
HS2	30,904,946	29,087,754	95.94	89.74	51.93	72.07
HS3	32,047,710	30,195,764	96.32	90.47	51.89	74.05

### Identification and annotation of DEGs

**Fig. 3:**
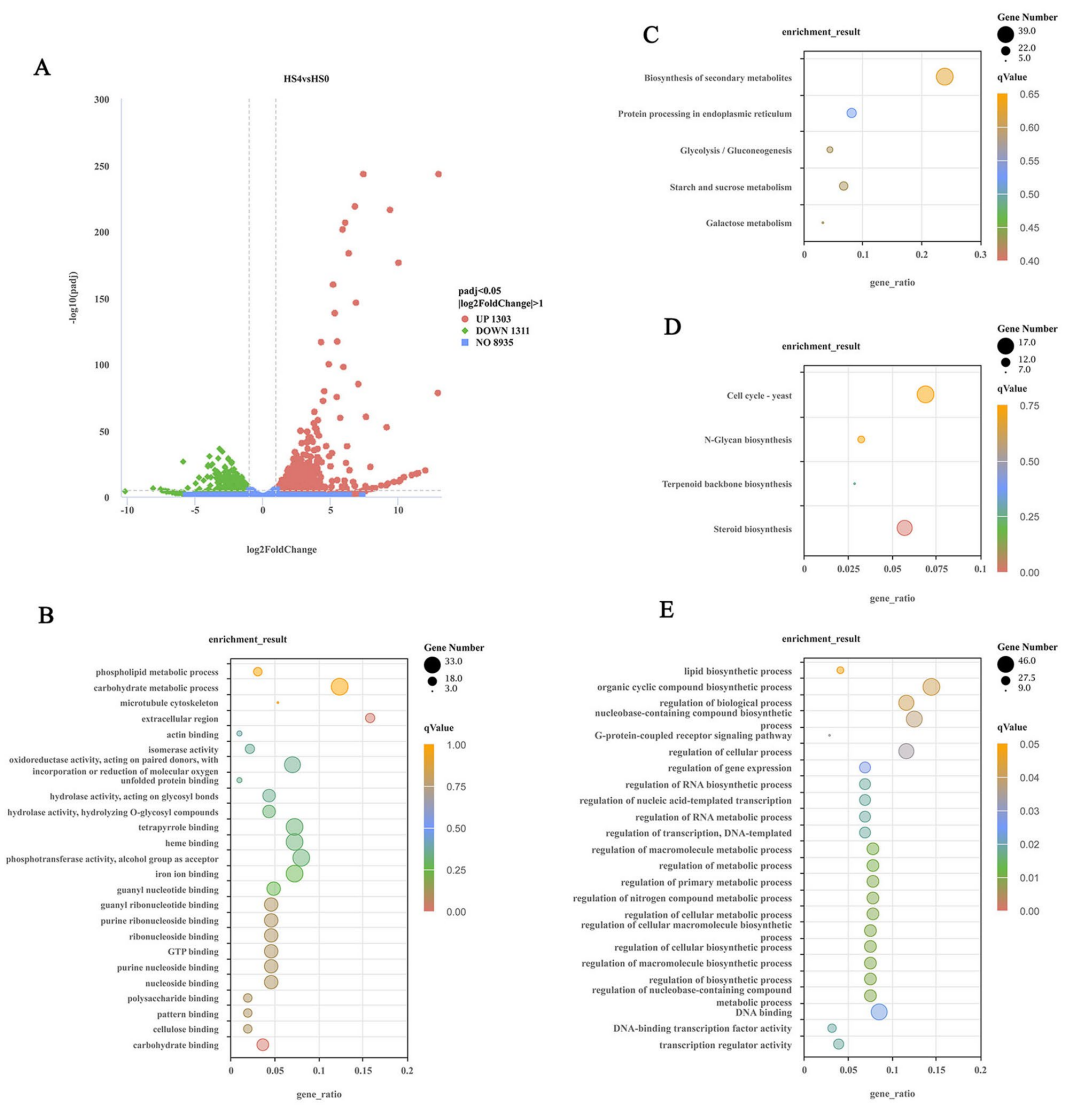
Analyses of differentially expressed genes (DEGs). **(A)** DEG distribution of the two treatment groups: heat shock (HS) vs. control (CK). The GO terms **(B)** and KEGG pathway **(C)** enrichment of up-regulated genes in PG46. The GO terms **(E)** and KEGG pathway **(D)** enrichment of down-regulated genes in PG46. Circle sizes represent gene counts, and circle colors indicate qValue

**Fig. 4:**
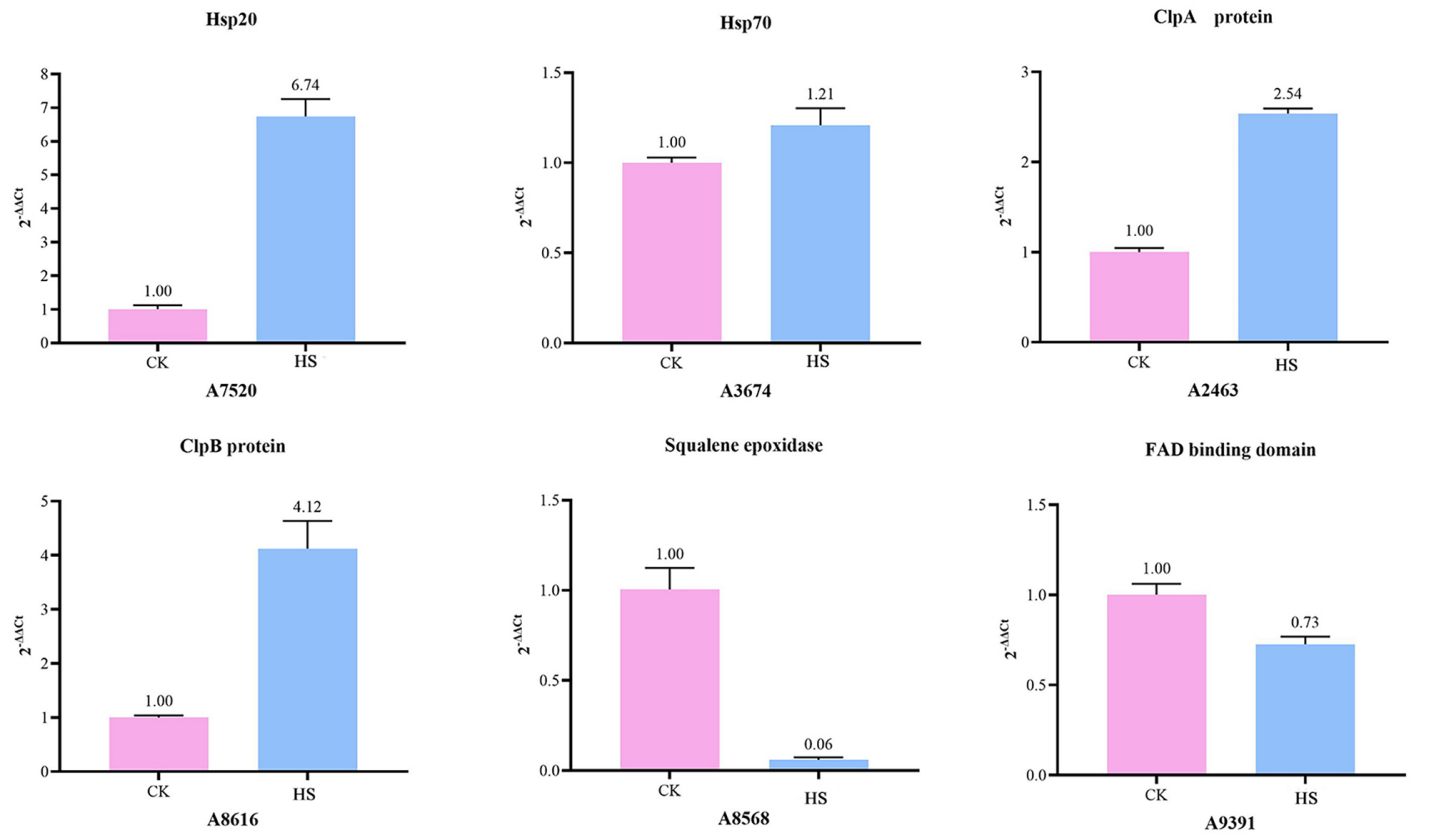
Validation of the gene expression levels of six DEGs

The DEseq method was used to analyze the significant DEGs under the screening criteria of expression difference multiple |log2 (fold change) | ≥ 1 and a false discovery rate ≤ 0.05. A total of 2,614 DEGs were identified between HS and CK groups, with 1,311 significantly down-regulated DEGs and 1,303 significantly up-regulated DEGs (Fig. 3A, Supplementary Table S2). Functional enrichment analysis showed that the down-regulated genes were enriched in 24 GO items (Fig. 3E) and 4 pathways (Fig. 3D), which are mainly related to regulation of biological process, regulation of metabolic process, organic cyclic compound biological process, regulation of translation and steroid biosynthesis, which indicated its carbohydrate metabolism may be impaired under high temperature stress, further affecting the synthesis of many compounds. Then further functional annotation of the DEGs was carried out, the results showed that 601 and 770 genes were annotated into Swiss-Prot and Pfam databases respectively, and 359 transcription factor (TF) family were identified. The analysis found that these genes were closely related to the growth and development of mushrooms, such as oxidoreductases and their binding domains (FAD/NAD), ATPase family, Sugar (and other) transporter, glycosyl hydrolase/ transferase family and Cyclin, which may be related to the prolonged growth period of PG46 after heat stress found in our previous research. The up-regulated genes were enriched in 25 GO items (Fig. 3B) and 5 pathways (Fig. 3C), which were mainly related to the carbohydrate-metabolic process, nucleoside binding, iron ion binding, photosynthesis activity, oxidoreduction activity, biosynthesis of secondary metabolites and glycolysis/gluconeogenesis, which may be involved in the regulation of high temperature tolerance of *P. giganteus*. In order to fully excavate the heat-resistant genes of *P. giganteus*, further functional annotation of the DEGs was carried out. The results showed that 524 and 674 genes were annotated into Swiss-Prot and Pfam databases respectively, and 331 transcription factor (TF) family were identified. The analysis found that the genes related to abiotic stress, such as heat shock protein, fungal protein kinase, serine/threonine-protein kinase, zinc finger protein and ubiquitin, were significantly up-regulated, which may be the key genes for high temperature tolerance of *P. giganteus* PG46.

In addition, LIM domain, SNARE protein, WD repeat protein, protein BMH1, serine/threonine-protein kinase, cytochrome c heme lyase, cyclin-1, metacaspase, and mitochondrial inner membrane protease related to the cell cycle and apoptosis were also identified as up-regulated DEGs; these genes may be helpful in alleviating heat stress damage. Interestingly, the encoding genes of laccase, alpha/beta-glucosidase, and xylanase related to lignocellulose degradation were also significantly up-regulated, suggesting that appropriate heat shock was conducive to substrate utilization.

### Validation of gene expression by qRT-PCR

According to the expression differences, six DEGs were selected for qRT-PCR analyses. Among them, four genes (two Hsps and two Clp proteins) were up-regulated and related to abiotic stress, while two genes (FAD-binding protein and squalene monooxygenase) were down-regulated and related to carbohydrate metabolism. Comparative analysis showed that the expression pattern detected by qRT-PCR was similar to the differential analysis results from the RNA-seq output (Fig. 4, Supplementary Table S3). This result suggested that our RNA-Seq data were reliable. In the future, these DEGs can be further explored as candidate genes for high temperature adaptation using functional genomics approaches.

## Discussion

The existing research on *P. giganteus* has mainly focused on its biological characteristics, nutritional value, active ingredients, and cultivation techniques, leaving limited insights into cellular and molecular aspects [20]. Based on the Pacific Biosciences Sequel platform, the whole genome sequencing of *P. giganteus* PG46 was carried out with its monokaryon obtained by protoplast isolation. Our analysis encompassed the genomic structure, genetic information, and phylogenetic evolution of *P. giganteus* were analyzed, as well as the exploration of functional genes related to heat stress. To obtain a high-quality genome of *P. giganteus*, the *de novo* assembly using SMRT Link and corrected with next generation sequencing data were employed. The assembly results showed that the genome size of PG46 was 40.11 Mb, consisting of 17 contigs with 2.89 Mb in N50 value, and the CEGMA and BUSCO values were 97.65% and 99.0%, respectively. Compared with previous reports [21], the contig number in our *P. giganteus* PG46 genome (17) was lower than that in *P*. *giganteus* zhudugu2 (27), indicating the higher quality of the newly assembled genomes in this study. The genome sizes of *Pleurotus* species have been reported to range from 34.5 Mb (*P. floridanus*) to 53.6 Mb (*P. eryngii* var. *eryngii*) [22], which aligns with our findings. Previous investigations of plants have indicated that differences in genome sizes between species primarily depend on the amplification of repetitive sequences [23], and this may also be the main cause of genome size variation between *Pleurotus* species. Out of the 13,054 coding genes, 10,695 (81.95%) genes were annotated in several functional databases mentioned. Functional genes related to abiotic stress were identified in GO annotation, including DNA repair, heat shock protein binding, transmembrane transport, zinc ion binding, protein kinase activity, and signal transduction [24–26]. These genes potentially play important roles in the high temperature adaptation of *P. giganteus*, but it was necessary to further verify whether they can be expressed under heat stress. In addition, some pathways related to abiotic stress were predicted, including mTOR, MAPK, PI3K-Akt, AMPK, and Rap1 signal pathways, shedding light on the high temperature resistance of *P. giganteus* [27–29].

The taxonomic status of *P. giganteus* has been a subject of controversy, initially described as *Le. giganteus* or *Pa. giganteus*. Recently, it was transferred to *Pleurotus* by Karunarathna et al. [13] based on morphological and phylogenetic ITS1-5.8 S-ITS2 rDNA sequence analyses. Our phylogenetic tree further supports this reclassification, affirming a closer evolutionary relationship between *P*. *giganteus* and other *Pleurotus* species. Meanwhile, we analyzed the evolutionary divergence times of *Pleurotus.* The divergence time between *P. ostreatus* and *P. cornucopiae* was estimated as 4.5 (2.5–6.9) Mya, while the divergence time between *P. gigantea* and *P. citrinopileatus* was estimated as 59.8 (35.0–64.0) Mya, which were consistent with previous findings [22]. In addition, we also found that the divergence time between *Pleurotus* and *Lentinus* was estimated as 153.9 (138.6–176.4) Mya, and they have a divergence time with *Pa. rudis* at (156.3–191.4) Mya, confirming the taxonomic status of *P. giganteus* on the genome level.

The assessment of the transcriptomic data showed that the average values of Q20, Q30 and mapping rates were 96.48%, 90.84% and 76.19%, respectively. In addition, the Pearson correlation coefficients of different replicates per group were greater than 91.5% (CK) and 92.2% (HS) in three replicates per group (Supplementary Figure S2). These parameters indicated that our RNA-seq data were dependable [30]. The enrichment analysis of DEGs showed that the 1,311 down-regulated DEGs were almost all associated with compound synthesis/metabolism processes, like oxidoreductases and their binding domains (FAD/NAD), ATPase family, Sugar (and other) transporter, glycosyl hydrolase/ transferase family and Cyclin, indicating that carbohydrate metabolism of PG46 under heat stress may be impaired, further affecting the synthesis of many compounds [31]. Oxidoreductase is a kind of enzyme that can catalyze the oxidation and reduction between two molecules. It participates in the synthesis and metabolism of many compounds and plays an important role in the growth and development of mushroom [32, 33]. Among them, FAD/NAD-binding domain-containing protein is an important oxidoreductase with a role in electron transfer, and was often classified as a photoreceptor in mushroom [34, 35]. Moreover, blue light was considered a main signal which prompts fruit body and pigment development, so researchers speculated that the FAD/NAD-binding domain-containing protein encoding gene might be involved in photomorphogenesis induced by blue light of mushroom. Leung et al. [36] used RNA fingerprinting by arbitrarily primed PCR (RAP-PCR) to identify differentially expressed genes in RNA populations from different stages of *Le. edodes*, the result showed that the genes involved in plasma membrane transport and metabolic pathways were significantly up-regulated, such as sugar transporter, cyclin, ATPase and glycosyl hydrolase/ transferase family, which may play important roles during the initiation of primordia and the formation of fruiting bodies. In our study, these genes were significantly down-regulated under heat stress, indicating that heat stress was not conducive to the growth and development of PG46, further explaining the prolonged growth period of PG46 under high temperature.

Notably, some genes directly related to abiotic stress were significantly up-regulated in the HS group, including heat shock protein (HSP), DnaJ protein, transcription factors, ubiquitin protease, DNA mismatch repair proteins, and zinc finger proteins [37–39], which further verified the results of whole genome sequencing. Therefore, it was speculated that these genes may be involved in the heat temperature tolerance of *P. giganteus*. Based on the molecular level and metabolite network, heat shock proteins (HSPs) were positively correlated with the organism’s thermotolerance [40–42]. In our study, the expression levels of some HSP genes in PG46 were strongly up-regulated after heat shock, including four *Hsp20*s, two *Hsp70*s, and one *Hsp90*. Similarly, after *A. bisporus* mycelia were exposed to high temperature, Hsp20, Hsp70, and Hsp90 proteins known to be potentially involved in *A. bisporus* thermotolerance also showed faster and more robust accumulation in the thermotolerant strain. Moreover, an *Hsp70* gene was located in the vicinity of the quantitative trait loci on linkage group II [43, 44]. Hsp20 comprises a major family of HSPs induced by elevated temperature, encoding a fascinating group of molecular chaperones associated with stress responses in a range of different species [45–49]. Fungi typically carry fewer *Hsp20* genes, usually less than five. Previous research has shown that the induced high expression of *Hsp20* genes not only enhances thermotolerance in organisms, but also contributes to the growth and survival at high temperatures [45, 47, 50]. *Hsp70* can protect nascent polypeptides and refold damaged proteins under heat stress conditions, and it has been demonstrated to be important for stress tolerance in almost all organisms [51]. Xu et al. [52] found that overexpression of *Hsp70* from *Hypsizygus marmoreus* enhanced the heat tolerance of tobacco, indirectly indicating the necessity of *Hsp70* for heat tolerance and recovery in *H. marmoreus*. Compared to *Hsp70*, *Hsp90* is involved in the final maturation of proteins. Therefore, during the mycelial growth of PG46, these genes may act as an “assembly line” for protein maturation under heat stress [43]. DnaJ proteins can promote protein translation, folding, unfolding, translocation, and degradation by stimulating the ATPase activity of chaperone proteins Hsp70s [53, 54] that play significant roles in organismal growth and development and resistance to abiotic and biotic stresses [37, 55, 56]. The relationship between DnaJ and thermotolerance has rarely been reported in basidiomycetes. In our study, Dnaj proteins were also significantly up-regulated under heat shock. According to Wang et al. [30], Le DnaJ not only regulates the thermotolerance of *Le. edodes* but also interacts with Letrp E, a ratelating enzyme in IAA biosynthesis, to regulate the thermotolerance of *Le. edodes* via meditating IAA biosynthesis. Therefore, the regulation of DnaJ under heat stress in *P. giganteus* needs further confirmation.

Heat signal transduction involves various transcription factors and protein kinases. Transcription factors (TF) from different families are significantly regulated in response to heat stress. Among them, Heat transcription factors (HSFs) are major TFs that mediate the heat stress response, and play a vital role in regulating HSP expression by recognizing a heat shock element located in the promoters of its target genes, thereby increasing the thermotolerance of plants and fungi [57, 58]. Moreover, hsfa2 knockout mutants of *Arabidopsis* are defective in thermometry, indicating that HSFs can regulate high temperatures tolerance of organisms by controlling the expression levels of HSPs [59]. Our results indicate that the expression levels of some TFs were upregulated in the thermotolerant strain PG46 after heat stress, suggesting that *P. giganteus* tolerance to heat stress may be positively controlled by HSFs. Protein kinases (PKs) are phosphotransferase that catalyzes protein phosphorylation and regulate various physiological mechanisms in organisms through phosphorylation and dephosphorylation, including metabolism, transcription, cell division, movement, and programmed cell death. PKs also play roles in the immune response and nervous system function [60]. Among the protein kinases,, serine/threonine-protein kinases (STPKs) are the most extensively studied and include cyclin-dependent kinase (CDK), mitogen-activated protein kinase (MAPK), DNA-dependent protein kinase, natto kinase, and protein kinase C [61]. In our study, many genes encoding STPK were significantly regulated in *P*. *giganteus* PG46 after heat stress, with both up-regulation and down-regulation observed. Therefore, we speculated that many kinds of protein kinases exist in *P. giganteus*, and different protein kinases perform various roles under heat stress conditions. In addition, some autophagy-associated protein kinases. such as BAG, HT1, EKC/KEOPS complex subunit, metacaspase, and tyrosine-protein kinase RIPK2, were also significantly up-regulated. These genes may play important roles in selective autophagy and selective macro autophagy as an adaptive mechanism to maintain cellular homeostasis in *P. giganteus* [62]. The activation of these pathways might aid in coping with heat stress and protecting the cellular integrity and function of *P*. *giganteus*.

## Conclusion

Our study successfully generated a high-quality genome of *P. giganteus* and identified key functional genes related to high-temperature adaptability. These genes hold promise as candidate targets for enhancing the high temperature resistance of *P. giganteus*. The findings of this research pave the way for further genetic modification of *P. giganteus* strains, facilitating the development of high-temperature resistant strains for the edible fungus industry.

## Methods

### Samples collection and DNA extraction

PG46, a typical high-temperature resistant strain of *P. giganteus*, was selected by our team through large-scale cultivation for five consecutive years. It can form fruiting bodies at 28–35 °C and is suitable for summer cultivation in hot areas. The biological transformation rate of this strain is 92.8%, with good commercial properties and neat mushroom production, making it suitable for facility cultivation. This strain was cultured on potato dextrose agar (PDA) medium at 28 ℃ for 7 days, and then stored under low temperature conditions of 4 ℃. The strain was maintained in the Environment and Plant Protection Institute at the Chinese Academy of Tropical Agricultural Sciences (EPPICATAS, Haikou, China). The monokaryon used for *de novo* genome sequencing, was isolated from the PG46 strain using a protoplast-derived method and was confirmed by microscopic and molecular identification [63]. The genomic DNA of the monokaryon was extracted with a novel plant genomic DNA extraction kit (CWBIO, Beijing, China), and the integrity, purity, and concentration of genomic DNA were examined by 0.6% agarose gel electrophoresis (100 V, 1.5 h) and Qubit 3.0 [64].

### Whole genome sequencing and assembly

The *de novo* genome sequencing was performed on Sequel II platform of Pacific Biosciences (PacBio), and the next generation sequencing was performed on an Illumina NovaSeq PE150 platform. The genome was assembled using SMRT Link v5.0.1 software and corrected with next generation sequencing data [65]. Finally, the accuracy and integrity of the assembled genome were examined using the Core Eukaryotic Genes Mapping Approach (CEGMA) and Benchmarking Universal Single-Copy Orthologs (BUSCO) [66, 67].

### Gene prediction and function annotation

Protein-coding genes in *P*. *giganteus* was predicted by integrating three approaches namely, *de novo* -based, homology-based, and RNA-Seq-based predictions. For the ab initio prediction, we used Augustus (v.2.7) [68], GlimmerHMM (v3.0.1) [69], and SNAP [70] to predict gene structures based on intrinsic features of the genome. In the protein homology-based prediction, we aligned protein sequences from closely related species, namely *Agaricus bisporus*, *P. ostreatus* and *Coprinopsis cinerea*, to the *P*. *giganteus* genome assembly using TBLASTN (E-value ≤ 1e-5) [71]. Subsequently, we identified the homologous genes with the help of GeneWise (v.2.4.1) [72]. For RNA-Seq-based predictions, the RNA-Seq reads were initially assembled using Cufflinks (v.2.2.1) with default parameters, and the resulting unigenes were then aligned to the repeat-masked assembly of *P*. *giganteus* using BLAT. Next, the gene structures were modeled based on the BLAT alignment results using PASA v2.4.1 [73]. To identify the protein-coding regions, TransDecoder v3.0.1 and GeneMarkS-T were utilized [74]. Finally, a consensus gene model was generated by integrating all the gene models obtained from the different methods and protein alignments using EVidenceModeler (v1.1.) [75], with different weights assigned to each prediction strategy’s outputs.

For non-coding RNA, the transfer RNAs (tRNAs) were predicted using tRNAscan-SE software [76]; the ribosomal RNAs (rRNAs) were predicted by *de novo* prediction using rRNAmmer software [77]; and the small RNAs (sRNAs), small nuclear RNAs (snRNAs) and micro RNAs (miRNAs) were predicted by Rfam database comparison and cmsearch program confirmation [78]. To identify repeats in the genome, we categorized them into scattered repeats and tandem repeats. Scattered repeats were predicted using RepeatMasker (version open-4.0.5) software [79], while tandem repeats were identified using tandem repeats finder (TRF) version 4.07b [80].

The protein sequences of the predicted genes were aligned with several functional databases by diamond comparison (E value < 1e-5), including the National Center for Biotechnology Information (NCBI) Non-Redundant Protein Database (Nr), Swiss-Prot [81], Gene Ontology (GO) [82], Kyoto Encyclopedia of Genes and Genomes (KEGG) [83], Eukaryotic Clusters of Orthologous Groups (KOG) [84], Transporter Classification Database (TCDB) [85] and Pfam database [86] The sequences with the highest alignment scores (default identity ≥ 40%, coverage ≥ 40%) were selected for functional annotation. Additionally, we performed specific annotations to further explore the functions of all coding genes. CAZYmes were identified using the CAZY database [87], secretory proteins were identified by SignalP (Version 4.1) [88] and TMHMM (Version 2.0c) tools [89], secondary metabolite gene clusters were predicted by antiSMASH − 4.0.2 progress (version 2.0.2) [90],and Cytochrome P450s proteins (CYPs) were identified by aligning the amino acid sequences to the fungal P450 database using BLAST software ((e-value ≤ 1 × 10^− 5^) [91].

### Phylogenetic and evolutionary analysis

The coding sequences of PG46 and nine other fungal species reported in NCBI were selected to perform a gene family analysis through OrthoMCL software (v.2.0.9) [92], including *P. ostreatus* PC9 [93], *P. citrinopileatus* HfpriPC05-1-Y1, *P. cornucopiae* CCMSSC00406 [94], *Panus rudis* PR-1116 ss-1 [95], *Lentinula edodes* L808, *Serpula lacrymans* S7.9 [96], *Coniophora puteana* RWD-64-598 SS2 [97], *Laccaria bicolor* S238N-H28 [98] and *Cop. cinerea* okayama7#130 [73]. The multiple sequence alignment was performed on the proteins of single-copy homologous genes using MUSCLE software [99]. The phylogenetic tree was constructed using the maximum likelihood method with RAxML software (1000 bootstrap replicates) based on the LG + I + G + F amino acid substitution matrix model selected by ProtTest software (v. 3.4) [100]. The neutral evolutionary rate and species divergence time of these species were estimated by the Markov Chain Monte Carlo algorithm using the MCMCTree program of the PAML software package [101]. The divergence times of *La. bicolor* and *Cop. cinerea* [59.3–108.4 million years ago (Mya)], *S. lacrymans* and *Con. puteana* [70.0–129.4 (Mya)], and the divergence time between the two groups (109.89–176.71 Mya) was used as the fossil time correction points [98, 102].

### Transcriptome sequencing and analysis

Comparative mRNA analyses of *P. giganteus* PG46 were performed between mycelia of heat treatment and control groups. In our previous research, the *P. giganteus* mycelia were inoculated on the PDA medium and cultured in darkness at 28 ℃ for 4 days. Afterward, the heat treatment group (HS) was subjected to 40 ℃ heat stress for 18 h, while the control group (CK) was kept at 28 ℃ for the same duration [8, 30, 103]. Each treatment was replicated three times, resulting in a total of six samples. Total RNA of the mycelia was extracted from each sample using Trizol Reagent (Tiangen, Beijing, China), following the manufacturer’s instructions. The concentration and integrity of RNA were determined using an Invitrogen Qubit 2.0 fluorometer and an Agilent Bioanalyzer 2100 system, respectively. Then, the mRNA sequencing library was established with high-quality RNAs using an NEBNext® Ultra™ Directional RNA Library Prep Kit [104]. Finally, the prepared libraries were subjected to sequencing on the Illumina Hiseq sequencing platform, and 150 bp paired-end reads were generated [105].

The raw datasets of the six samples underwent initial processing using FastQC and were stored in the fastq file format [106]. In order to obtain high-quality clean reads, the reads containing adapters or poly-N and low-quality reads of raw data were removed using Trimmomatic (v.0.33) [107], and the error rate, Q20, Q30, and GC content of the clean data were calculated. Then, the clean reads were mapped to the reference genome of *P. giganteus* PG46 by HISAT2 (v.2.10) with the default settings [108]. The mapped sequences were assembled and annotated using StringTie software [109] and functional databases. The feature Counts tool was used to count the mapped reads of each gene [110]. The expression level of each gene was evaluated according the fragments per kilobase of transcript per million fragments sequenced (FPKM) value [111]. Differentially expressed genes (DEGs) between two samples were identified by DESeq2, and the *P* value was adjusted by the Benjamini and Hochberg method [112]. Genes with |log2 (fold change) | ≧ 1 and a false discovery rate (FDR) ≦ 0.05 were considered as statistically significant DEGs [105]. Functional annotation of the DEGs was performed using the GO, KEGG, Pfam and SwissProt databases. Based on the annotation results, functional enrichment analyses of DEGs were performed by cluster Profiler with an adjusted *P*-value < 0.05 [113].

### Quantitative real-time PCR (qRT-PCR) validation of DEGs

To validate the RNA-seq results, six DEGs were selected and analyzed by qRT-PCR. The primer pairs were designed using Primer 5.0 (Supplementary Table S1). β-tubulin was selected as the internal reference gene (F: 5’ – CTTTCTTGCATTGGTACACGC – 3’; R: 5’ – TCGCCTTCTTCCTCATCGGCA – 3’).

Total RNA was isolated as described above. According to the manufacturer’s instructions, RNA was reverse-transcribed using a TransScript® All-in-One First-Strand cDNA Synthesis SuperMix for qPCR (One-Step gDNA Removal) kit (TransGen Biotech, Beijing, China). Then, 2 X RealAtar Green Fast Mixture with ROX (GenStar BioSolutions, Beijing, China) was used to perform qRT-PCR on the QuantStudio 6 Flex Real-Time PCR System (Thermo Fisher Scientific, Massachusetts, USA). The reaction system and conditions of qRT-PCR were conducted according to Fu et al. [29]. The relative expression of genes was determined using the 2^− ΔΔCt^ method [114].

### Electronic supplementary material

Below is the link to the electronic supplementary material.

Supplementary Material 1

Supplementary Material 2

Supplementary Material 3

Supplementary Material 4

Supplementary Material 5

## Data Availability

The raw sequencing datasets generated for this manuscript have been uploaded to GenBank. The genome raw sequencing data are associated with NCBI BioProject: PRJNA896531 and BioSample: SAMN31552355 (https://submit.ncbi.nlm.nih.gov/subs/wgs/SUB12237535/overview); The transcriptome raw sequencing data are associated with NCBI BioProject: PRJNA898808, BioSample: SAMN31627222 (CK) and SAMN31627223 (HS), SRA: SRR22207284 (CK) and SRR22207283 (HS) (https://submit.ncbi.nlm.nih.gov/subs/sra/SUB12255315/overview).
