# Supplementary figures and images for: Integrative analysis of genome and transcriptome reveal the genetic basis of high temperature tolerance in pleurotus giganteus (Berk. Karun & Hyde)
Source: BMC Genomics. 2023 Sep 18;24:552. doi: 10.1186/s12864-023-09669-8 (PMC10506213; doi:10.1186/s12864-023-09669-8)

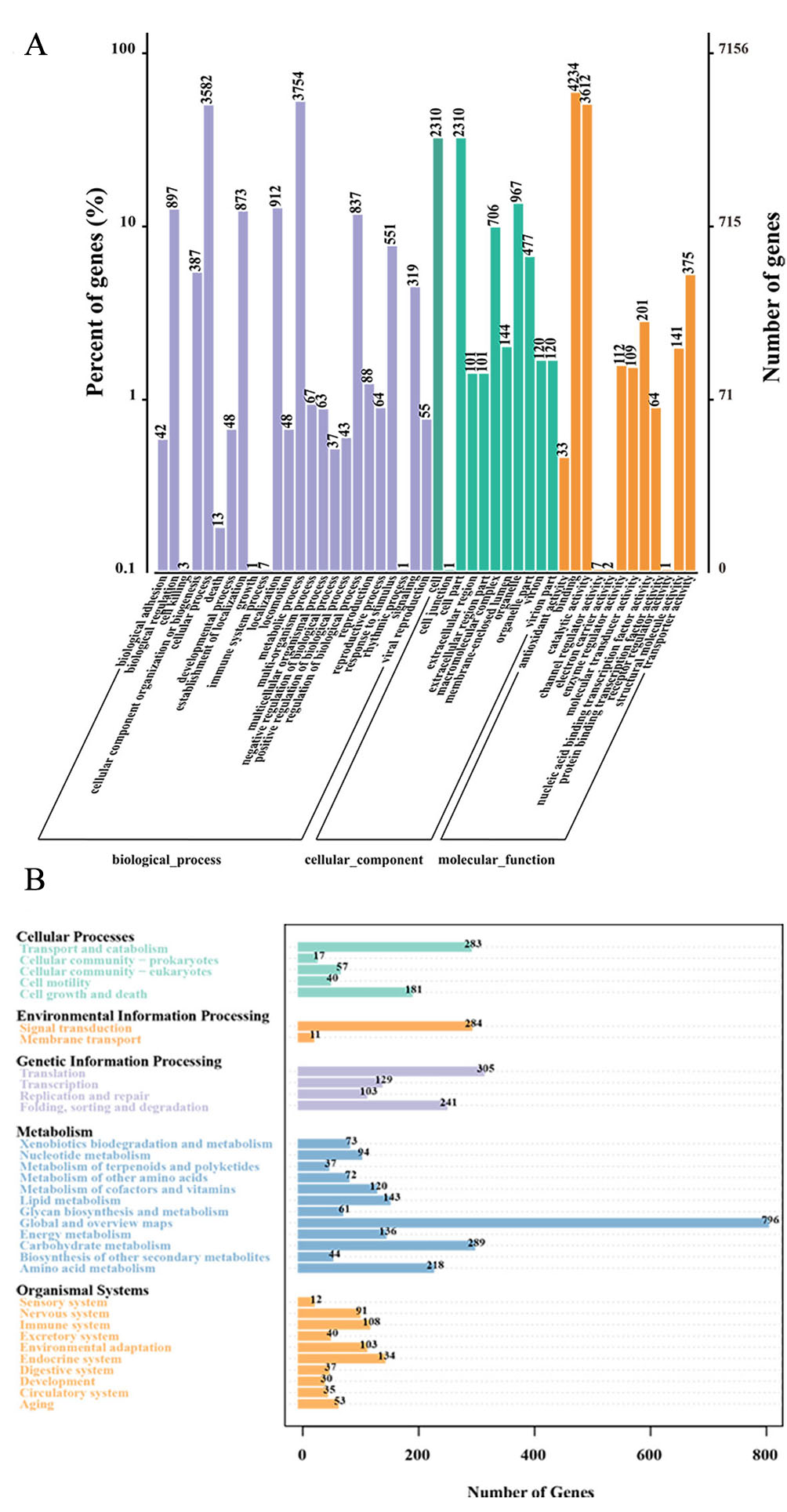

Supplement: Supplementary file 4 — Supplementary Material 4 [file 12864_2023_9669_MOESM4_ESM.jpg]

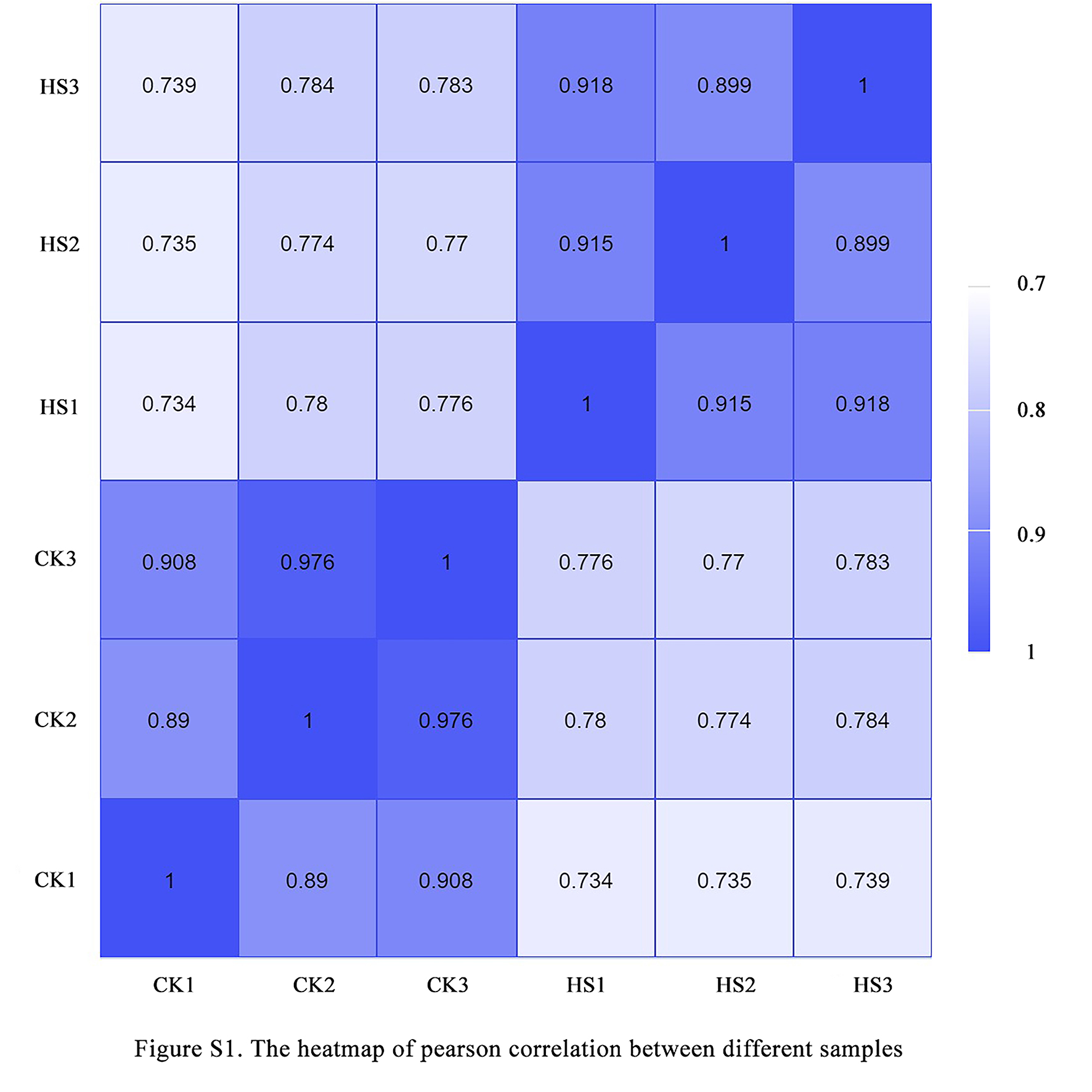

Supplement: Supplementary file 5 — Supplementary Material 5 [file 12864_2023_9669_MOESM5_ESM.jpg]
